# Outcomes after selective nerve root blockade for lumbar radicular pain from lumbar disc hernia or lumbar spinal stenosis assessed by the PROMIS-29 – a prospective observational cohort study

**DOI:** 10.1007/s00701-024-06196-7

**Published:** 2024-07-25

**Authors:** Caroline Karlsson, Erik Carlsson, Josefin Åkerstedt, Petrus Lilja, Christoffer von Essen, Pedram Tabatabaei, Johan Wänman

**Affiliations:** 1 Department of Diagnostics and Intervention, Umeå University, Umeå, Sweden; 2 Department of Molecular Medicine and Surgery, Stockholm Sports Trauma Research Center, Karolinska Institute, Stockholm, Sweden; 3 Department of Clinical Sciences, Neurosurgery, Umeå University, Umeå, Sweden

**Keywords:** Selective nerve root block, Lumbar disc herniation, Lumbar spinal stenosis, Foraminal stenosis, Lumbar radicular pain, Patient related outcome measures

## Abstract

**Purpose:**

Selective nerve root blocks (SNRB) are used both as a therapeutic and diagnostic tool for lumbar radicular pain. Most studies evaluate the effect of SNRB simply by its relation to pain reduction. It is well known that pain is associated with other factors such as depression, anxiety, inactivity and sleeping disorders, but these patient-related outcomes are seldom evaluated. This study evaluated the influence of SNRB on pain-related outcomes including depression, anxiety, fatigue, pain interference, activity and sleep.

**Methods:**

One hundred three patients with lumbar radicular pain were treated with a SNRB. Patient-reported outcome measures (PROMs) were assessed with the PROMIS-29 for 12 weeks (84 days) following the SNRB. Patients were stratified based on their pain reduction at the 14-day follow up as responders (≥ 30% pain reduction) and non-responders (< 30% pain reduction). Post-treatment duration was estimated with the Kaplan–Meier analysis with return to baseline as an event. A paired t-test was used to compare pre- and post-treatment responses at specific time intervals.

**Results:**

Forty-four percent (*n* = 45) of the patients were responders and showed significant improvement in all parameters throughout the 84-days follow-up, the exception was sleep that lost significance at day 70. The mean post-treatment duration among responders was 59 (52–67) days. Non-responders showed significant improvements in pain interference and pain intensity until day 35 and in ability for social participation until 21-day.

**Conclusion:**

SNRB can improve pain intensity, pain interference, physical function, fatigue, anxiety, depression, sleep disturbance and the ability to participate in social roles.

**Supplementary Information:**

The online version contains supplementary material available at 10.1007/s00701-024-06196-7.

## Introduction

Lumbar radicular pain (LRP) is defined as pain from the lower back that radiates along the course of a particular lumbar nerve. LRP has an estimated prevalence of 3–5% [1, 24]. Selective nerve root blockade (SNRB) acts by reducing the inflammation and the intensity of pain by injecting cortisone often in combination with local anaesthetic in close proximity to the affected nerve root. Several studies have demonstrated SNRB as a safe and cost-effective treatment compared to spinal surgery in patients with LRP [20–22, 24] but the usefulness has been questioned [2]. Most previous studies addressing the effect of SNRB have used the visual analog scale (VAS) and/or the Oswestry disability index (ODI) as outcomes [9, 11, 15, 24]. Long term pain increases the risk for mental, physical, and social health disorders [7, 17] but such consequences have rarely been assessed for patients with LRP either before or after SNRB. A study of such pain-related consequences would enable a more extensive evaluation of the consequences of LRP and the usefulness of SNRB. The Patient Reported Outcomes Measures Information System (PROMIS-29) assesses mental health, physical health and social health through seven domains with four items each rated on a five-point scale including fatigue, pain intensity, pain interference, anxiety, ability to participate in social roles, depression, physical function and sleeping disorders [16]. The PROMIS-29 has shown good validity, reliability and usefulness for patients undergoing spine surgery [16]. The present study used the PROMIS-29 in order to evaluate patients with LRP in relation to mental, physical and social health before and after SNRB.

## Methods

### Study design

This prospective cohort study included 103 patients that underwent SNRB due to LRP at Umeå University Hospital between 2021 and 2023.

The inclusion criteria were patients ≥ 18 years of age with unilateral or bilateral radicular leg pain with or without back pain after conservative treatment and a MRI verified corresponding nerve root compression.

### Radiographic assessment

An MRI was performed in all patients prior to the SNRB. The level of root compression was evaluated and the type of compression was categorized as disc herniation or spinal stenosis. The disc herniations were categorized according to the Michigan State University (MSU) Classification, which reports size and location in three precise increments. The disc herniation is described simply as 1–2-3 according to size and A-B-C according to location, all taken from a single measurement of the intra-facet line [13]. The spinal stenosis was graded as 0 (no stenosis), 1 (mild stenosis), 2 (moderate stenosis) and 3 (severe stenosis) according to the Lee system [12].

### Nerve block technique

**Fig. 1:**
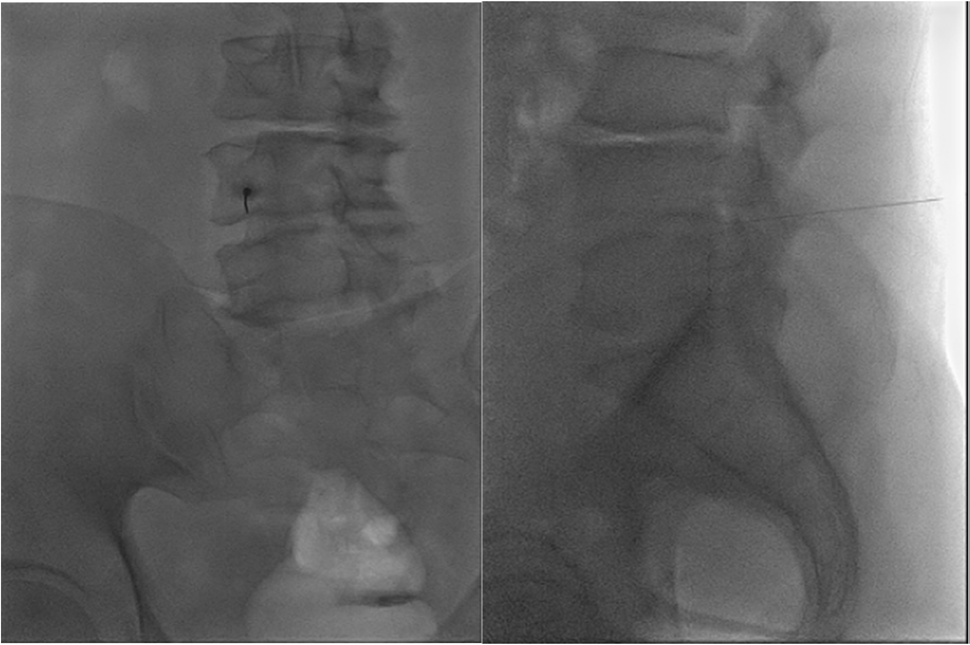
Illustration of the final position of the needle. The needle was positioned near the targeted nerve root under fluoroscopic guidance. Both an anterio-posterior (left) and a lateral (right) fluoroscopic image were taken, followed by the injection of 1 ml Marcaine (2.5 mg/ml) and 1 ml Depo-Medrol (40 mg/ml) at the target site

All blockades were performed at the neurosurgical department by the same neurosurgeon, who also managed the fluoroscopy during the intervention. All patients underwent the same procedure. The procedures took place in an interventional neuroradiology hybrid operating room with biplanar fluoroscopy (Azurion 7B20, Philips Medical Systems, Best, Netherlands). Patients were placed in prone position and prepared with sterile draping before the blockade was performed. Depending on the patient's body size, either an 88 mm or a 120 mm, 22-gauge spinal needle was used. The needle was advanced and positioned near the targeted nerve root under fluoroscopic guidance. The target point was just lateral to the neural foramen. When this point was reached, both an anteroposterior and a lateral fluoroscopic image were taken and saved for confirmation and journal keeping, followed by the injection of 1 ml Marcaine (2.5 mg/ml) and 1 ml Depo-Medrol (40 mg/ml) at the target site (Fig. 1), contrast media was not injected prior to the blockade.

### Patient-reported outcome measures

In this study, the PROMIS-29 was self-administered at admission for SNRB (baseline) and 14 days after followed by every week for 12 weeks (84 days) after the SNRB. The primary outcomes were pain-related consequences, evaluated by the PROMIS-29. Fatigue, pain intensity, pain interference, anxiety, ability to participate in social roles, depression, physical function and sleep disturbance were analysed at baseline and changes in each variable compared to baseline on the following days day; 14, 21, 28, 35, 42, 56, 70 and 84 days after the SNRB.

Number of patients that were admitted for spinal surgery or additional SNRB 6 months after the initial 84 days evaluation of the SNRB was recorded.

### Statistics

Descriptive statistics for continuous variables are presented as means with a 95% CI and categorical data are presented as numbers and percentages. Histogram and QQ plots were used for the verification of normal distribution. The student’s t-test was used for comparison between means for variables with normal distribution. Categorical variables were analysed by Chi2-test. A paired t-test was used out to compare pre- and post-treatment responses at 14, 21, 28, 35, 42, 56, 70 and 84 days. The data were further stratified based on patients that had at least 30% pain reduction (responders) and patients with less than 30% (non-responders), a subgroup analyse of patients with at least 50% pain reduction was also performed. A posttreatment duration was estimated using Kaplan–Meier analysis with return to baseline as an event. A *p* < 0.05 was considered to be significant. We used SPSS (IBM SPSS Statistics for Mac, Version 26.0, Armonk, NY: IBM Corp. USA) for statistical analyses.

### Ethics

Written informed consent was obtained from all participants. The study complies with the ethical principles of the Helsinki Declaration and was approved by the Swedish Ethical Review Authority (Dnr 2023–01061-01).

## Results

### Patient characteristics

**Fig. 2:**
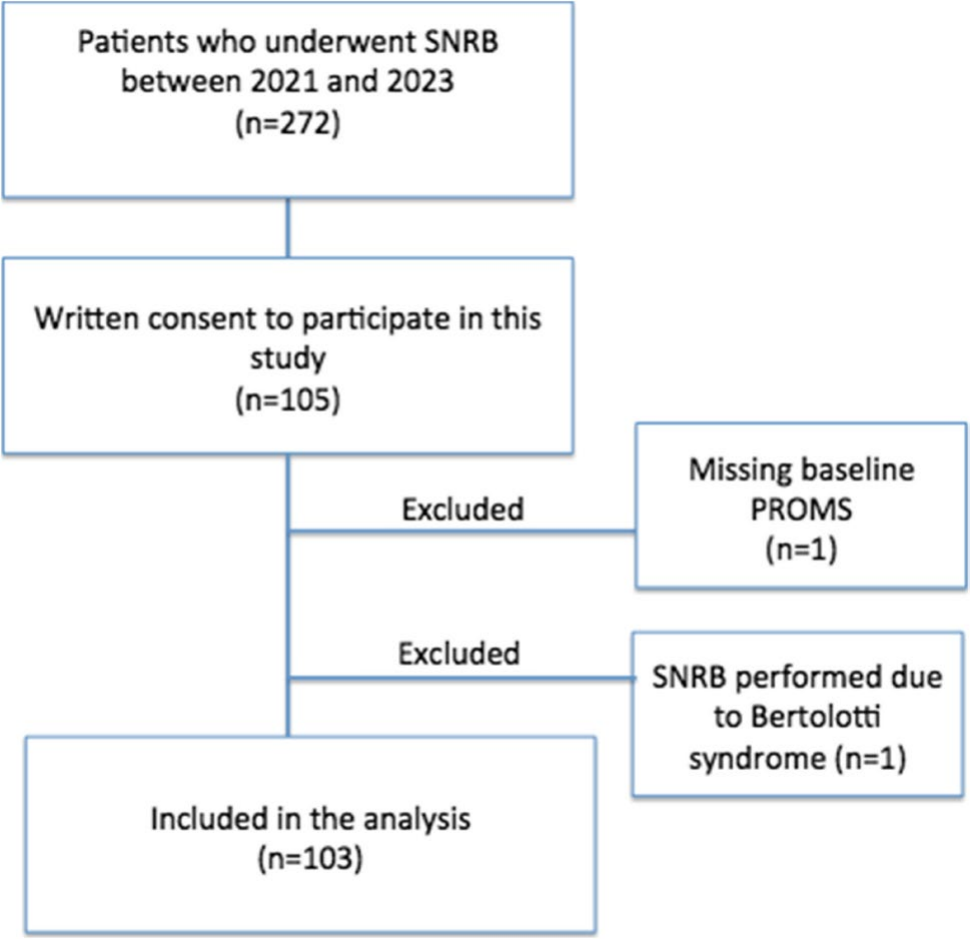
Flowchart of the patients

We included 103 patients (64 female and 39 males) with a mean age of 64 (61–66) years. The most commonly affected nerve root was L5 (*n* = 49). Patient characteristics are presented in Table 1. A flowchart is presented in Fig. 2.

**Table 1:** Patient characteristics and baseline PROMIS-29 before SNRB

	Responders*	Non responders**	*P*-value
Number of patients	45	58	
Age	64 (59.8–68.2)	63(59.5–66.3)	0.69
Gender			0.065
Men	12 (27%)	27 (46%)	
Women	33 (73%)	31 (54%)	
Duration			0.41
0–3 month	3 (7%)	3 (5%)	
3–6 months	4 (9%)	2 (7%)	
> 6 months	38 (84%)	53 (92%)	
Comorbidity			
Hypertension	26 (58%)	26(44%)	0.24
Diabetes	6 (13%)	7 (12%)	1.0
Cardiovascular	11 (24%)	11(19%)	0.48
Asthma	7 (16%)	13(22%)	0.46
COPD	2 (4%)	2 (3%)	1.0
Psychiatric disorders	7 (16%)	8 (14%)	0.79
Opioid use	16 (36%)	21 (36%)	1.0
Baseline PROMS-29			
Pain intensity	7.3 (6.9–7.8)	7.0 (6.5–7.5)	0.27
Pain interference	66.5 (64.7–68.3)	66.4 (64.3–68.5)	0.66
Fatigue	56.5 (52.9–60.1)	57.1 (54.1–60.2)	0.73
Physical function	35.8 (34.1–37.6)	36.8 (35.0–38.7)	0.30
Anxiety	57.0 (54.1–60.0)	55.9 (52.9–58.8)	0.56
Depression	56.3 (53.4–59.2)	55.8 (53.1–58.4)	0.82
Sleep disturbance	55.6 (53.2–57.9)	56.3 (54.3–58.3)	0.21
Ability to participate in social roles	40.5 (38.5–42.6)	40.0 (37.7–42.2)	0.16

* Patients with ≥ 30% pain reduction 14 day after the SNRB

** Patients with < 30% pain reduction 14 day after the SNRB

### Categorization of root compression

Sixty-four patients underwent SNRB due to lumbar spinal stenosis (LSS) and 39 due to a lumbar disc hernia (LDH). There was a significant difference between patients with LDH and LSS with a higher proportion of patients categorized as responders after the SNRB in the spinal stenosis group (*p* = 0.013). No differences were seen between different MSU categories (*p* = 0.81) or grades of LSS (*p* = 0.14), (Table 2).

**Table 2:** MRI classification of LDH and LSS in patients stratified as responders* and non-responders**

Type of compression	Responders	Non-responders	P-value
Disc herniation according to MSU***			
1A	1	1	
1AB	1	1	
1B	2	9	
1C	2	3	
2AB	1	1	
2B	1	6	
2C	1	4	
3AB	0	1	
3B	2	2	
3C	0	0	
Total	11	28	0.81
Central, recess or lateral Stenosis****			
Unable to classify	3	0	
Grade I	3	6	
Grade II	13	15	
Grade III	15	9	
Total	34	30	0.14

* Patients with ≥ 30% pain reduction of the NRS

** Patients with < 30% pain reduction of the NRS

***Michigan State University Classification. Classified according to size (1–3) and location from the intra-facet line from central to lateral (A-C) measures in axial projections

****Classified according to Lee et al. graded from mildly to severe stenosis (I–III), measured in axial projections

### Baseline parameters

#### Physical health

At baseline, the pain interference assessment demonstrated a mean score of 66.5 (65.1–67.8), fatigue scores demonstrated a mean score of 56.8 (54.5–59.2) and physical function a score of 36.4 (35.1–37.7).

#### Mental health and social ability

The mean score at baseline was 56.4 for anxiety (54.3–58.5), 56.0 (54.1–57.9) for depression and 56.0 (54.5–57.4) for sleep disturbance. The mean score for the ability to participate in social roles and activities was 40.2 (38.7–41.8).

### Outcomes after blockade

#### Responders, pain reduction and duration

**Fig. 3:**
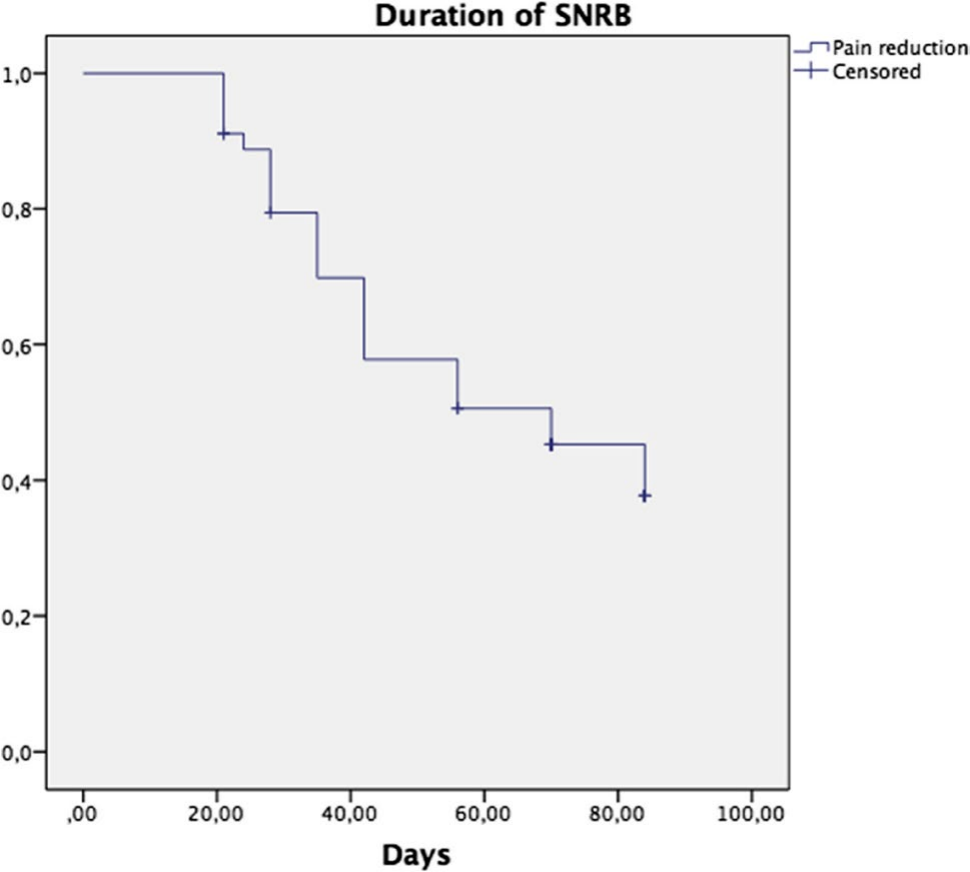
Kaplan–Meier curve illustrating the duration of pain relief after selective nerve root blockade in the responder group with return to baseline as an event. Patients that had had ≥ 30% pain reduction of the NRS were classified as responders

Forty-five patients (44%) were classified as responders with a pain reduction of at least 30% at the first follow up after the SNRB (at 14 days), 33 patients had a pain reduction of at least 50%. The mean duration among responders was 59 (52–67) days (Fig. 3). The mean reduction in pain intensity at the 14 days evaluation was 4.4 (3.8–4.9) (*p* < 0.001) for responders and 0.6 (0.3–0.9) (*p* = 0.001) for the non-responders. Table 1 show baseline data including physical health, mental health and social ability for the responders and non-responders respectively; there were no significant differences between these groups.

### Change in outcomes after SNRB

#### Physical health

The mean changes in physical function, fatigue, pain interference and pain intensity are presented in Table 3. At 14 days, the mean change in physical function score for the responders was -5.3 (-6.9- -3.7) (*p* < 0.001) and the changes remained significant for the full 84 days. The non-responders did not significantly improve in physical function at 14-days. At 14 days, the mean change in fatigue score for the responders was 6.0 (3.9–8.2), (*p* < 0.001), and the changes remained significant for the full 84 days. The non-responders did not significantly improve in the fatigue score at 14 days. At 14 days, the mean change in pain interference for the responders was 8.7 (6.6–10.9), (*p* < 0.001), and the changes remained significant for the full 84 days. The non-responders reported a mean change in pain interference of 1.0 (0.06–2.0) (*p* = 0.038) at 14 days, and the changes remained significant up to 35 days.

**Table 3:** Mean change in physical health and pain intensity after SNRB in responder* and non-responder** group

	Physical function	*P*-value	Fatigue	*P*-value	Pain interference	*P* value	Pain intensity	*P*-value
Responders* Days after SNRB								
14	-5.3 (-6.9- -3.7)	< 0.001	6.0 (3.9–8.2)	< 0.001	8.7 (6.6–10.9)	< 0.001	4.4 (3.8–4.9)	< 0.001
21	-5.4 (-6.9- -3.8)	< 0.001	5.9 (3.3–8.6)	< 0.001	8.4 (6.3–10.5)	< 0.001	4.0 (3.4–4.6)	< 0.001
28	-5.0 (-6.9- -3.1)	< 0.001	6.0 (3.4–8.6)	< 0.001	7.1 (4.7–9.4)	< 0.001	3.4 (2.7–4.1)	< 0.001
35	-5.0 (-7.0- -2.9)	< 0.001	4.6 (2.0–7.1)	0.001	7.5 (5.0–9.9)	< 0.001	3.4 (2.6–4.1)	< 0.001
42	-4.6 (-6.7- -2.6)	< 0.001	5.4 (2.8–8.0)	< 0.001	7.3 (4.9–9.8)	< 0.001	3.2 (2.5–3.9)	< 0.001
56	-4.3 (-6.5–-2.2)	< 0.001	3.4 (0.6–6.3)	0.02	5.7 (3.0–8.4)	< 0.001	2.6 (1.8–3.4)	< 0.001
70	-4.3 (-6.5–-2.0)	< 0.001	3.8 (1.0–6.5)	0.009	5.6 (2.8–8.4)	< 0.001	2.6 (1.8–3.4)	< 0.001
84	-4.5 (-6.7–-2.3)	< 0.001	4.2 (1.6–6.8)	0.003	5.4 (2.5–8.3)	0.001	2.6 (1.7–3.4)	< 0.001
Non-Responders** Days after SNRB								
14	-0.8 (-1.6–0.15)	0.10	0.1 (-1.8–2.1)	0.89	1.0 (0.06–2.0)	0.038	0.6 (0.9–0.9)	0.001
21	-1.1 (-2.1- 0.0)	0.05	1.1 (-0.9–3.1)	0.29	3.0 (1.7–4.3)	< 0.001	0.7 (0.3–1.1)	0.001
28	-0.7 (-1.8–0.46)	0.24	0.5 (-1.7–2.7)	0.66	2.1 (0.7–3.5)	0.003	0.9 (0.4–1.4)	0.004
35	-1.0 (-2.4–0.30)	0.12	-0.6 (-3.0–1.8)	0.63	2.2 (0.5–3.9)	0.012	0.9 (0.4–1.4)	0.001
42	-0.4 (-1.8–1.0)	0.57	0.3 (-2.0–2.7)	0.77	1.5 (-0.3–3.4)	0.10	0.5 (-0.01–1.1)	0.056
56	0.5 (-2.0–0.9)	0.48	-0.4 (-2.9–2.0)	0.73	1.7 (0.2–3.2)	0.024	0.5 (-0.04–1.0)	0.069
70	-0.4 (-1.8–1.0)	0.54	-0.6 (-3.0–1.8)	0.61	2.2 (0.4–4.0)	0.020	0.5 (-0.02–1.0)	0.059
84	-0.1 (-1.4–1.2)	0.86	-2.2 (-4.4–0.09)	0.059	1.5 (-0.2–3.3)	0.085	0.4 (-0.1–1.0)	0.12

* Patients with ≥ 30% pain reduction 14 day after the SNRB

** Patients with < 30% pain reduction 14 day after the SNRB

Patients with at least 50% pain reduction after the SNRB reported a mean change of 6.2 (-8.3- -4.2) (*p* < 0.001) in physical function, 6.4 (3.6–9.1) (*p* < 0.001) in fatigue and 7.3 (11.2–3.8) (*p* = 0.001) in pain interference and the changes remained significant for the full 84 days.

#### Mental health and social ability

The mean changes in anxiety, depression, sleep and ability to participate in social roles and activities are presented in Table 4.

**Table 4:** Mean change in mental and social participation after SNRB in responder* and non-responder group**

	Anxiety	P-value	Depression	*P*-value	Sleep	*P*-value	Ability to participate	*P*-value
Responders* Days after SNRB								
14	3.8 (1.6–6.1)	0.001	4.1 (1.9–6.2)	< 0.001	4.6 (2.7–6.5)	< 0.001	-3.8 (-5.9- -1.8)	0.001
21	5.4 (3.0–7.8)	< 0.001	4.9 (2.5–7.2)	< 0.001	5.7 (3.2–8.1)	< 0.001	-5.9 (-8.3- -3.4)	< 0.001
28	5.9 (3.1–8.7)	< 0.001	4.3 (1.7–6.9)	0.002	4.1 (1.6–6.6)	0.002	-5.0 (-7.4- -2.7)	< 0.001
35	4.6 (2.1–7.0)	0.001	3.4 (1.0–5.7)	0.006	4.2 (1.5–7.0)	0.003	-6.2 (-8.8- -3.6)	< 0.001
42	6.3 (3.7–8.9)	< 0.001	4.0 (1.4–6.5)	0.003	4.1 (1.4–6.8)	0.004	-5.5 (-7.9- -3.1)	< 0.001
56	5.1 (2.3–7.9)	0.001	3.0 (0.3–5.7)	0.031	3.1 (0.4–5.9)	0.026	-3.6 (-6.3- -0.9)	0.011
70	5.3 (2.3–8.2)	0.001	4.4 (1.2–7.5)	0.008	2.6 (-0.3–5.4)	0.074	-4.6 (-7.5- -1.8)	0.002
84	5.9 (2.8–9.0)	< 0.001	3.6 (0.6–6.6)	0.019	3.0 (-0.2–6.2)	0.069	-3.9 (-6.9- -0.9)	0.013
Non-Responders** Days after SNRB								
14	-1.7 (4.0–0.6)	0.15	-0.4 (-2.2–1.3)	0.64	1.2 (-0.1–2.6)	0.069	-2.7 (-4.2- -1.3)	< 0.001
21	0.7 (-1.6–3.0)	0.54	0.5 (-1.2–2.2)	0.56	1.6 (0.08–3.2)	0.039	-2.2 (-3.6- -0.9)	0.002
28	0.2 (-2.2–2.6)	0.87	0.1 (-1.8–2.0)	0.94	1.2 (.0.5–2.9)	0.16	-1.6 (-3.3–0.15)	0.072
35	0.6 (-1.9–3.1)	0.64	0.5 (-1.4–2.5)	0.58	1.3 (-0.6–3.2)	0.19	-1.4 (-3.2–0.4)	0.14
42	0.9 (-1,9–3.7)	0.53	0.3 (-2.1–2.6)	0.83	1.3 (-0.3–2.9)	0.11	-1.1 (-2.9–0.7)	0.23
56	-0.2 (-3.0–2.6)	0.89	0.2 (-2.0–2.4)	0.86	0.9 (-0.8–2.6)	0.29	-1.9 (-3.5–0.3)	0.018
70	0.3 (-2.5–3.0)	0.86	-0.02 (-2.3–2.2)	0.98	0.9 (-0.8–2.7)	0.29	-1.6 (-3.2- 0.09)	0.063
84	-0.6 (-3.5–2.3)	0.69	0.1 (-2.4–2.5)	0.95	0.5 (-1.4–2.3)	0.60	-0.3 (-2.1–1.5)	0.72

* Patients with ≥ 30% pain reduction 14 day after the SNRB

** Patients with < 30% pain reduction 14 day after the SNRB

At 14 days, the mean change in the anxiety score for the responders was 3.8 (1.6–6.1) (*p* = 0.001), and the changes increased over time up to 5.9 (2.8–9.0) (*p* < 0.001) at 84 days. The non-responders did not significantly improve in anxiety score.

At 14 days, the mean change in depression for the responders was 4.1 (1.9–6.2) (*p* < 0.001), and the changes remained significant for the full 84 days. The non-responders did not significantly improve in depression.

At 14 days, the responders reported a significant improvement in sleeping disturbance with a mean change of 4.6 (2.7–6.5) (*p* < 0.001), and the improvements remained significant up to 56 days. The non-responders did not significantly improve in sleep disturbance.

At 14 days, the mean change in the ability to participate in social roles and activities for the responders was -3.8 (-5.9- -1.8) (*p* = 0.001), and the improvement remained significant for the full 84 days.

The non-responders showed a significant improvement in the ability to participate in social roles and activities with a mean change of -2.7 (-4.2- -1.3) (*p* < 0.001) at 14 days, and the changes remained significant at 21 days (*p* = 0.002).

Graphs for the duration of outcomes for each of these variables are presented in the supplementary.

Patients with at least 50% pain reduction after the SNRB reported a mean change at the 14 day evaluation of 4.9 (2.0–7.7) (*p* = 0.002) for anexiety, 4.9 (2.2–7.8) (*p* = 0.001) for depression, 6.4 (3.6–9.1) (*p* < 0.001), 5.2 (2.8–7.6) (*p* < 0.001) for sleep disturbance and, -5.7 (-9.8- -1.7) (*p* = 0.007) in ability to participate and the changes remained significant for the full 84 days.

### Surgical treatment and repeated SNRB

Within the first 6 months after the 84 days evaluation of the SNRB, 30 patients were admitted for surgical treatment (13 patients with disc herniation and 17 patients with LSS, *p* = 0.46). Twenty-two patients (7 with LDH and 15 with LSS) were admitted for a repeated SNRB (*p* = 0.51).

## Discussion

In this prospective study, outcomes after SNRB were assessed with the PROMIS-29. Patients that were classified as responders reported significantly improved outcomes in pain intensity, pain interference, physical function, fatigue, anxiety, depression, sleep disturbance and the ability to participate in social roles.

To our knowledge, patients with LRP have not been previously evaluated with the PROMIS-29 after SNRB. The baseline parameters for the PROMIS-29 categories were similar to Pope et al. [19] who evaluated the mean scores in respective categories for the PROMIS-29 in 19,546 patients with chronic pain. This implies that the PROMIS-29 is a useful tool for evaluation of patients with LRP. The PROMIS-29 has been evaluated in relation to responsiveness and for estimation of minimal clinically important difference (MCID) for each variable in patients with chronic lower back pain [19]. The authors suggested a 5-points improvement in the PROMIS-29 scales as a reasonable indication of a MCID in patients with chronic lower back pain. In our cohort, the patients that were classified as responders improved significantly in all outcomes. Physical function, fatigue, pain interference, anxiety, sleep disturbance and the ability to participate in social roles and activities all improved more than 5 points at some time point of the study period after the SNRB; only depression had less than a 5 point improvement but was 4.9 at the 14-day evaluation. This emphasizes new clinically relevant perspectives for SNRB as a treatment alternative for patients with LRP. The significant effect for all the parameters in the PROMIS-29 for patients that were classified as responders supports the use of SNRB as an effective treatment for LRP with a clinically important effect not only for pain reduction but also for pain interference, depression, fatigue, anxiety, sleeping disorders and social participation. Pain interference had the largest reported improvements of all variables in our study. However, less than half of the patients were classified as responders. Responses after SNRB vary widely in the literature and therefore the clinical utility of a SNRB has been questioned [19]. We evaluated responders at 14 days and some patients may have responded to the SNRB but with a shorter duration of the effect. The significant effects for the responders compared to the non-responders in all outcomes variables emphasize the importance of patient selection for SNRB. In a systematic review by Nagington et al. [14], 42 potential clinical and radiological characteristics were evaluated for a positive outcome of SNRB. The study showed conflicting results and highlighted the uncertainty regarding predictive characteristics for suitable patients for SNRB. LDH is associated with inflammation [5] and corticosteroids injected by the SNRB may consequently have an additional effect for LDH, in contrast we found a higher proportion of responders in the LSS group compared to patients with LDH, this highlight SNRB as an alternative for RP for LSS patients. However, no associations were found between the proportion of responders and the severity of nerve root compression for either the LDH or the LSS group. In contrast, previous studies have reported an association between milder nerve root compression and positive outcomes after SRNB in patients with sciatica due to lumbar disc herniation [3, 6]. Patients that were categorized as non-responders did not improve in physical function, fatigue, anxiety or depression. However, they report significant improvements in pain interference, sleep disturbance and the ability for social participation although none of these variables had a mean improvements of 5 points or more, which indicates that these changes may not be clinically relevant [10].

The mean duration of 59 days for the responders was shorter compared to other studies [8, 11, 15, 20–22, 24]. However, many of the previous studies used up to seven SNRBs during the follow-up period. To postpone or avoid surgical intervention, Viswanathan et al. suggested that in case of an initial good pain relief, the patient could benefit from repeated injections at regular intervals [23].

Nerve blockades for sciatica can be performed using different methods [18]. In the present study, SNRB was used which has a similar approach as transforaminal epidural injection. Previous studies have used different approaches such as transforaminal or interlaminar injections [6, 9, 11, 15, 24]. However, the transforaminal approach is the most common and due to the proximity of injection to the affected nerve root this approach has shown to be the most effective [4, 24]. Because of a similar approach as transforaminal injection we compare our results with studies that used SNRB or transforaminal injection.

To quantity the benefit of a given treatment PROMs is an important tool. ODI assesses the level of disability and is a frequently used questionnaire in the field of spine surgery. It contains ten domains regarding pain intensity and pain during activities of everyday life. In interpretation, the result is calculated as the percentage of total points with high scores indicating a more severe disability. Also, an equation has been presented to derive ODI score from the PROMIS-29 to facilitate comparison between different studies [16]. A significant clinical effect on the ODI and VAS has previously been reported for SNRB [9, 20, 23]. The present study adds to previous literature with new insights on the clinical effect on other pain related outcomes. In particular, the statistically and clinically significant effect on sleeping, fatigue, anxiety, depression, ability to participate in social roles provides new knowledge about potential benefits after SNRB in patients with LRP.

### Strengths and limitations

The prospective design with a rigorous monitoring for PROMs during the 12 week follow-up are the main strengths of our study. Furthermore, all SNRBs were performed in the same manner by the same doctor. The main limitation of the study is that no adjusted analyses were performed for other variables that may have an effect on the outcomes.

## Conclusion

SNRB improved pain intensity, pain interference, physical function, fatigue, anxiety, depression, sleep disturbance and the ability to participate in social roles, however, fewer than half of the patients were categorized as responders.

## Supplementary Information

Below is the link to the electronic supplementary material.

Supplementary file1 (PDF 64 kb)

## Data Availability

The datasets generated during and/or analysed during the current study are available from the corresponding author on reasonable request.
