# Supplementary material for: Outcomes after selective nerve root blockade for lumbar radicular pain from lumbar disc hernia or lumbar spinal stenosis assessed by the PROMIS-29 – a prospective observational cohort study
Source: Acta Neurochir (Wien). 2024 Jul 25;166(1):306. doi: 10.1007/s00701-024-06196-7 (PMC11272668; doi:10.1007/s00701-024-06196-7)
Supplement: Supplementary file 1 — Supplementary file1 (PDF 64 kb) [file 701_2024_6196_MOESM1_ESM.pdf]

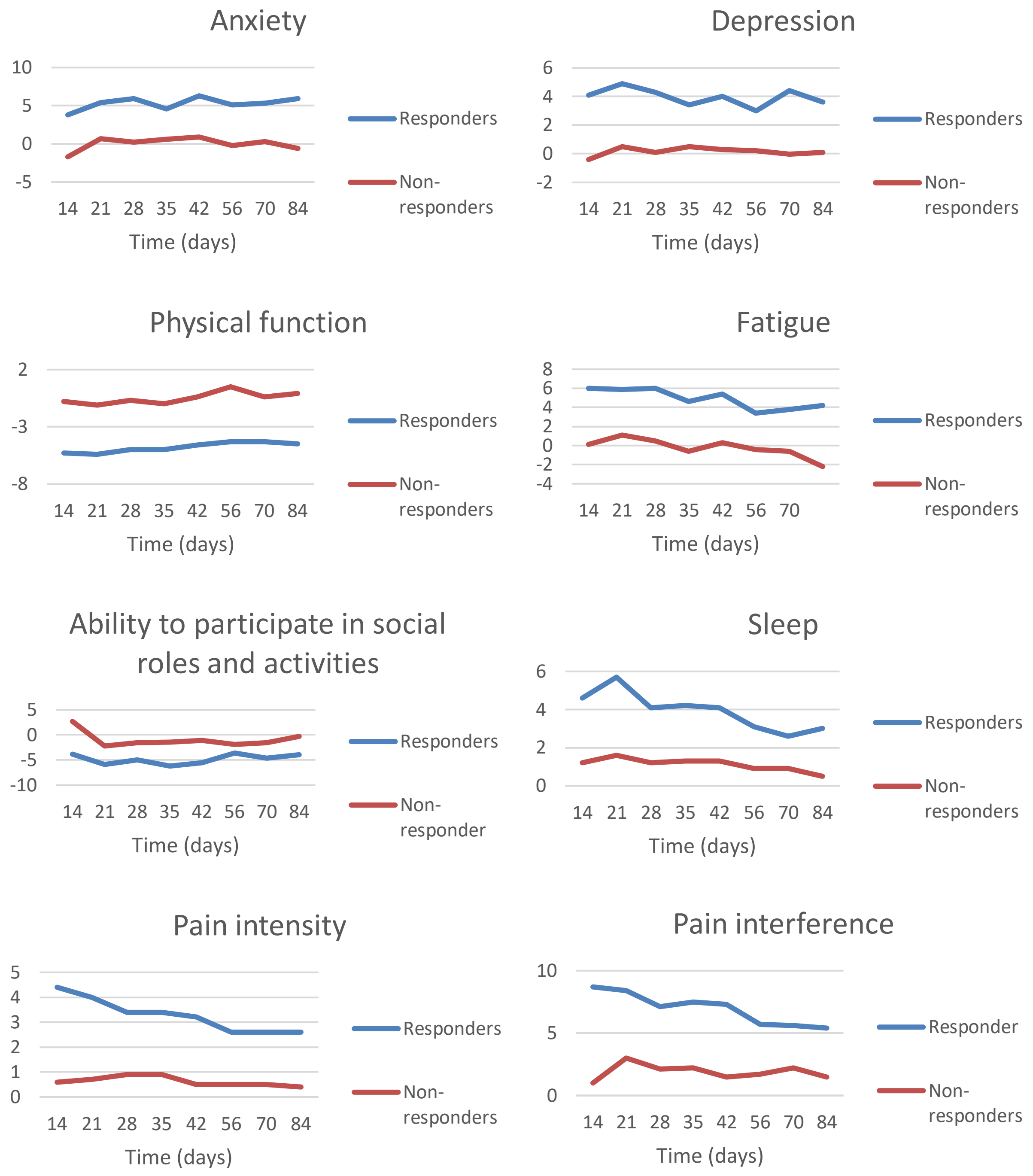

Supplementary. Line graph illustrating mean change in each category compared to baseline, in responder and non-responder group. Responders had at least 30% pain reduction and non-responders had less than 30%. X-axis represents time in days; y-axis, represents mean change.
